# Follicular dendritic cell sarcoma of gastrointestinal tract with two emerging distinct subtypes: a case report and systemic review

**DOI:** 10.1186/s13000-022-01246-z

**Published:** 2022-08-08

**Authors:** Hongxing Gui, Jigisha Chaudhari, Rifat Mannan

**Affiliations:** Department of Pathology and Laboratory Medicine, Pennsylvania Hospital of the University of Pennsylvania Health System, 801 Spruce Street, 10th Floor Spruce building, Philadelphia, PA 19107 USA

**Keywords:** Follicular dendritic cell sarcoma, Gastrointestinal tract, Spindle cell, Birt-Hogg-Dubé syndrome

## Abstract

**Background:**

Follicular dendritic cell sarcoma (FDCS) is a rare neoplasm of mesenchymal origin. FDCS of gastrointestinal tract (GI) are exceedingly uncommon.

**Case presentation:**

We report the first case of classic type FDCS in a 34-year-old male with Birt-Hogg-Dubé syndrome, which presented as a mass at the ileo-cecal junction. He received no further treatment after resection and remained disease free for 3.5 years. We further analyze and review the clinical and pathologic findings of 33 cases of GI tract FDCS reported in the literature.

**Conclusions:**

There are two distinct subtypes of FDCS in the GI tract: the classic type occurs in relatively younger patients (mean = 45.3 years) without Epstein-Barr virus (EBV) association, and behaves more aggressively; the inflammatory subtype presents as colonic polypoid tumor in older patients (mean = 60.7 years) and is EBV positive. The clinical outcome in the latter group appears favorable although mortality rate is not necessarily low.

## Background

Follicular dendritic cell sarcoma (FDCS) is an uncommon neoplasm derived from antigen presenting follicular dendritic cells, which normally reside in primary and secondary lymphoid follicles and function as part of accessory immune system interacting mainly with B lymphocytes [1, 2]. FDCS may occur in lymphoid follicles, either nodal, extra-nodal sites or both [3]. Morphologically, FDCS bears resemblance to its normal counterpart with spindled or ovoid shaped cells, mimicking metastatic poorly differentiated carcinoma and other mesenchymal neoplasms such as gastrointestinal stromal tumor (GIST). Because of its rarity, extranodal FDCS has been misdiagnosed in a significant number of cases [3–5]. With fewer than 50 cases reported in the literature, gastrointestinal (GI) tract FDCS especially poses a great diagnostic challenge with broad differential diagnoses.

Although FDCS is difficult but still recognizable by its histology and immunophenotype, the cause and molecular pathogenesis remains largely unknown. FDCS is categorized into classic/conventional type and inflammatory EBV positive variant. Classic FDCS is not associated with EBV infection and a subset of cases coexist with or are preceded by hyaline vascular Castleman disease [1, 6, 7]. By contrast, inflammatory EBV+ FDCS consistently demonstrates an association with EBV infection and typically arises in the liver and/or spleen, however more cases have recently been reported in the GI tract [8–10].

We present an unusual case of FDCS in the GI tract in a male patient with Birt-Hogg-Dubé syndrome. The tumor arose adjacent to ileocecal valve without lymph node or distant metastasis. He was also found to have papillary thyroid carcinoma concurrently. He received no further treatment after resection of the tumors and remains disease free for 3.5 years after the initial diagnosis. We further analyze and review all the GI tract FDCS reported in the literature to uncover the distinctive clinicopathological features of the two subtypes.

## Case presentation

**Fig. 1:**
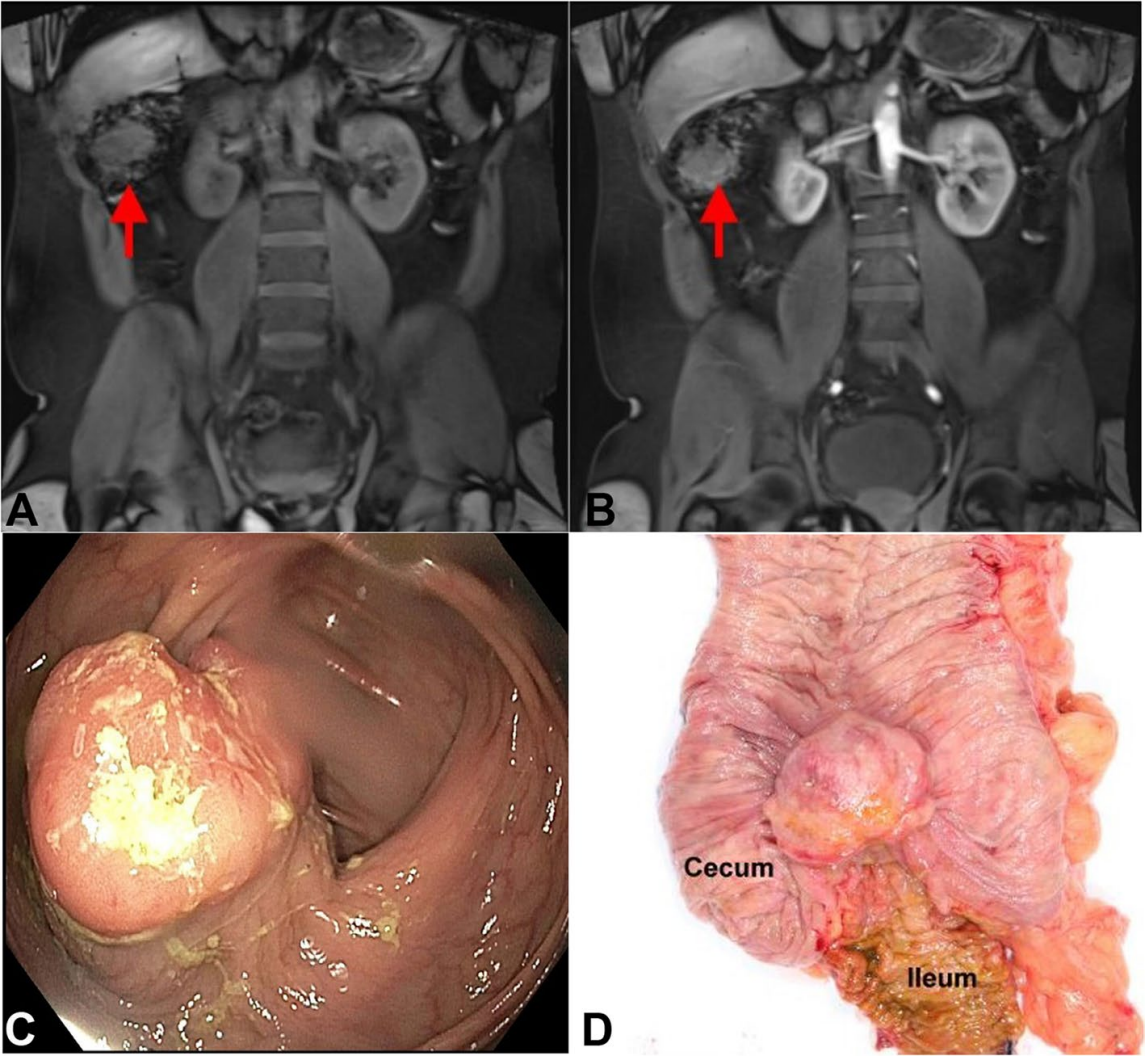
**A** Unenhanced MRI showed relative low T1W signal (arrow) in the tumor. **B**. Arterial phase showed early heterogeneous enhancement (arrow). **C** The tumor was masqueraded under colonoscopy as edematous and inflamed ileocecal valve with overlying frond-like/villous mucosa. **D** Right colectomy showed a tan ulcerated polypoid mass close to ileocecal valve protruding into the mucosal surface

A 34-year-old man with no significant past medical history presented with mild iron deficiency anemia (Hemoglobin 13.2 g/dL) and hematochezia for a month. He had no significant weight loss and had no family history of colorectal cancer, polyps or inflammatory bowel disease. The patient underwent a colonoscopy, which showed ileocecal valve to be edematous and inflamed with overlying frond-like/villous mucosa. (Fig. 1C). A biopsy was taken, which was reported as ulcerated mucosa, and was negative for malignancy.

An abdominal MRI scan was performed, which demonstrated a 4.1 × 4.0 × 2.9 cm mass with enhancement, located just distal to the ileocecal valve. This submucosal mass was thought to be most likely a gastrointestinal stromal tumor (Fig. 1A, B). Thereafter the patient underwent right hemicolectomy.

**Fig. 2:**
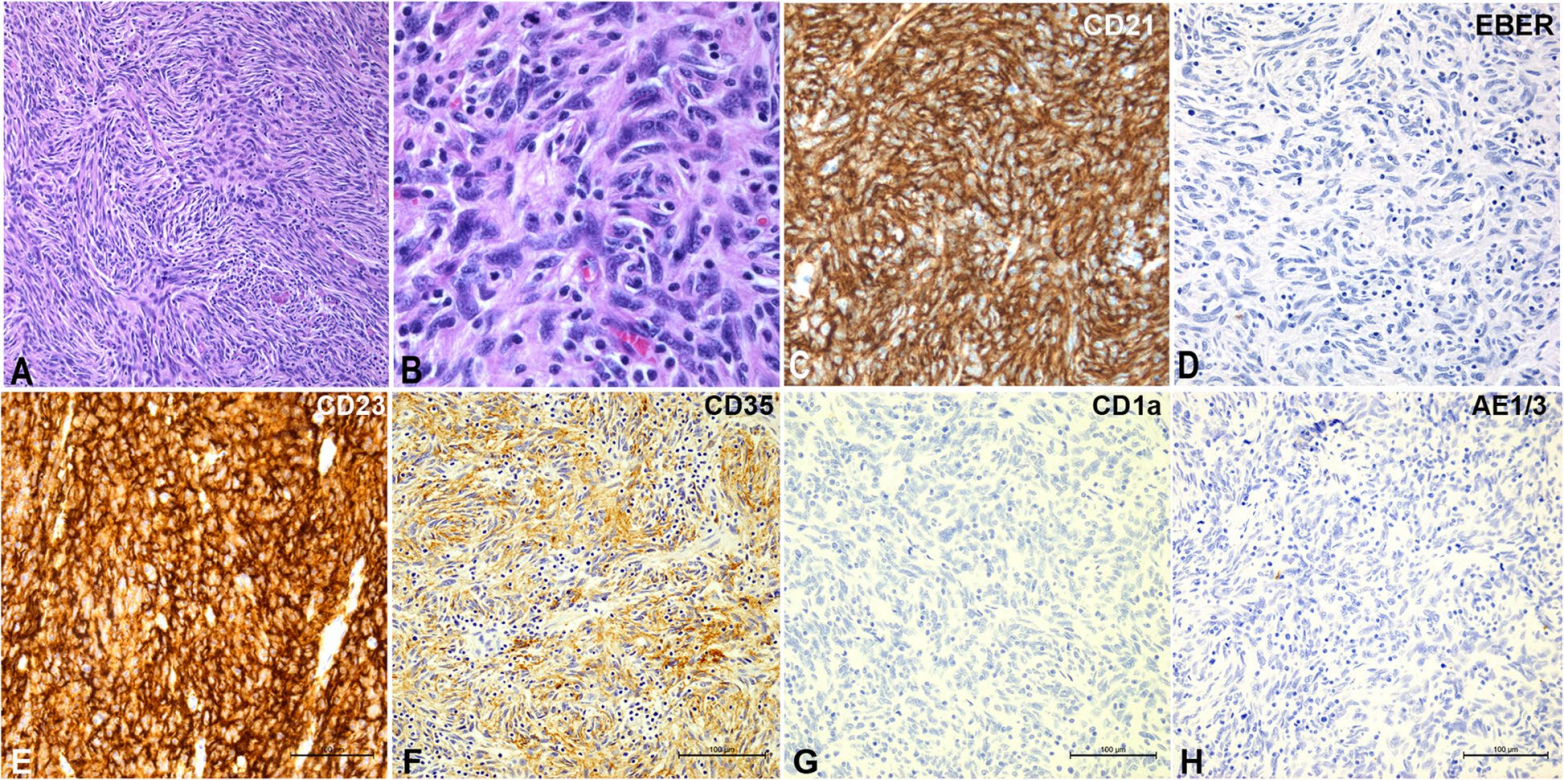
Microscopic views demonstrate a mass composed of spindle cells arranged in fascicles and storiform patterns in a background of prominent lymphocytic infiltrates (**A**). The neoplastic cells had eosinophilic cytoplasm, ovoid to spindled nuclei, vesicular chromatin and small nucleoli (**B**). The tumor cells stained positive for CD21 (**C**), CD23 (**E**), CD35 (**F**), while negative for EBER(D), CD1a (**G**), and cytokeratin AE1/3 (**H**)

Grossly, there was a tan ulcerated polypoid submucosal mass close to ileocecal valve measuring 4.5 × 3.7 × 2.8 cm (Fig. 1D). Microscopy revealed a multi-lobated mass centered in the muscularis propria, with extension into subserosa and focally extending into colonic mucosa. The lesion was composed of ovoid to spindle cells arranged in fascicles, storiform and whorled architecture (Fig. 2A). The tumor cells were focally clustered and intimately mixed with small lymphocytes (Fig. 2B). The neoplastic cells had eosinophilic cytoplasm with indistinct cell border, ovoid to spindled nuclei, vesicular chromatin and small nucleoli. There was mild cytologic atypia, but no coagulative necrosis was identified. Mitotic figures were found up to 10 per 10 high power fields. Immunohistochemical studies showed that tumor cells were positive for CD21 (Fig. 2C), CD23 (Fig. 2E), and CD35 (Fig. 2F), while negative for CD1a (Fig. 2G), C-kit, DOG1, SMA, AE1/3 (Fig. 2H), S100, SOX10, CD34, ALK, and EBER (Fig. 2D). A diagnosis of extra-nodal FDCS, French Federation of Cancer Centers Sarcoma Group (FNCLCC) grade 1 was rendered. No post-operative chemoradiation was administered given its morphological features and stage.

During the workup of FDCS, he was also found to have a left thyroid nodule measuring 4.5 cm. Total thyroidectomy revealed a macrofollicular variant of papillary thyroid carcinoma, which was encapsulated with multiple foci of capsular invasion. Furthermore, the patient had family history of melanoma, osteosarcoma and spontaneous pneumothorax. Therefore, a genetic testing of 47 hereditary cancer genes including *TP53*, *BAP1* and *FLCN* was performed, demonstrating a pathogenic heterologous germline mutation in the *FLCN* gene (c.1021delC). Thus the patient was diagnosed to have Birt-Hogg-Dubé syndrome. He was found later by a dermatologist to have fibrofolliculomas, skin tags, and multiple benign nevi on cheeks and upper back, but no renal tumor and pneumothorax was found.

After the initial presentation, the patient had been followed up for 3.5 years with no signs of local recurrence and distant metastasis of FDCS.

## Discussion

Follicular dendritic cell sarcoma is a low to intermediate grade neoplastic proliferation of spindled to ovoid cells derived from follicular dendritic cells. In 1986, Monda et al. first reported cases with features suggestive of dendritic reticulum cell differentiation [11]. Extranodal FDCS was first reported in oral cavity [12] and comprise 58% of 343 reported cases [3]. Among extranodal FDCS, liver and spleen are the most common organs involved, presenting typically as inflammatory EBV+ subtype of FDCS [13–15]. The EBV+ inflammatory FDCS is seen most commonly in young to middle aged adults (mean age 54.5 years) with a female predilection (2.2:1) [14]. Gastrointestinal FDCS is exceedingly rare, accounting for 5.2% among 343 cases [3].

We searched the literature for reported cases of gastrointestinal FDCS and found 32 cases [5, 6, 8–10, 16–35]: 20 cases of classic/conventional type and 12 cases of inflammatory EBV+ type. The clinicopathological features of two types are compared in Table 1.

**Table 1:** Two subtypes of gastrointestinal FDCS

	Classic FDCS	Inflammatory EBV+ FDCS	*P* value
Case number	*n* = 21	*n* = 12	
Mean age (median)	45.3 years (43)	60.7 years (58.5)	0.0011
Gender (M:F)	1:1.1	1:1	
Location	Upper GI: Lower GI = 1.1	Colon, polypoid mass	
Size (mean, cm)	7.8	3.0	0.0002
EBV	All negative	All positive	
OS (mean)	14.6 months	36.4 months	0.12
DFS (mean)	10.2 months	36 months	0.071
Recurrence	38% (8/21)	8.3% (1/12)	0.087
Mortality rate	14.3% (3/21)	16.7% (2/12)	0.601

*Abbreviations*: *DFS* disease free survival, *EBV* Epstein-Barr virus, *FDCS* follicular dendritic cell sarcoma, *GI* gastrointestinal, *OS* overall survival

Classic gastrointestinal FDCS occurs more commonly in middle age adults with no gender predominance (mean age = 45.3 years, M:F = 1:1.1). It is equally found in both upper and lower GI tract (upper: lower = 11:10), and presents as larger masses measuring 7.8 cm in average. Classic GI FDCS was found to recur after an average of 10 months with a recurrence rate of 38% (8/21). Three patients died of disease and mean overall survival time was 14.6 months.

In contrast, the inflammatory EBV+ type FDCS in the GI tract all presents as colonic polyps/masses. It occurs in older patients (mean age = 60.7 years) with no predilection of gender (M:F = 1:1), which is unlike inflammatory EBV+ type FDCS arising from liver and spleen with female predominance [14]. Follow-up ranged from 5 to 122 months and mean overall survival was 36.4 months. One patient had recurrence and two patients died of disease or paraneoplastic syndrome, with a mortality rate of 16.7% (2/12).

Because of its rarity and close resemblance to other common spindle cell tumors, FDCS can be misdiagnosed in 18.6 to 58% of the cases depending on anatomic locations of the tumors [3–5]. The initial diagnosis may be made erroneously as undifferentiated carcinoma, lymphoma, inflammatory myofibroblastic tumor (IMT), gastrointestinal stromal tumor (GIST) and many others. Amongst 21 cases of classic GI FDCS, 24% (5/21) were first diagnosed as GIST and 24% (5/21) had initial impression of a sarcoma. One case was thought as carcinoma and another was diagnosed as lymphoma. In the past, inflammatory EBV+ FDCS in GI tract was very rare, with only one confirmed case [27]. This might be due to under-diagnosis as it usually presents as fewer neoplastic cells embedded in a rich lymphoplasmacytic background, which can easily mislead into a diagnosis of reactive lesion, IMT or lymphoma. Another possible explanation of rarity is that inflammatory EBV+ FDCS only arises in Southeast Asia, either due to specific virulent EBV strain and/or genetic predispositions.

Conventional FDCS consists of spindled to ovoid cells which form fascicles, storiform arrays, whorls, diffuse sheets and vague nodules. The tumor cells are intermixed with small lymphocytes which can be seen aggregating around vessels. The tumor stains for one or more of the FDC markers including CD21, CD23, CD35, D2-40, clusterin, CXCL13, FDC-secreted protein and Serglycin [1, 36]. Cytokeratin, CD1a, langerin, lysozyme and myeloperoxidase stain is negative, while variable pattern of staining is seen for EMA, S100 and CD68. However, it is not uncommon that one or multiple FDC markers can be lost in the GI FDCS (24%, 8/33 cases). FDCS may present with variable histomorphology and immunohistochemistry therefore both morphological features and immunohistochemistry play pivotal roles in diagnosis of FDCS. The morphological pattern of FDCS may overlap with ectopic meningioma, myoepithelial tumor, IMT, thymoma and GIST, while osteosarcoma like pattern was occasionally described [37, 38]. As the neoplastic cells in FDCS often show focal staining of dendritic cell markers, a wide panel with 3 to 5 FDC markers is recommended. Most importantly, a consideration of FDCS in the differential diagnosis might be necessary when reviewing spindle cell lesions in polypoid GI masses. In terms of differential diagnosis for EBV+ inflammatory FDCS, one group of IMT with EBV association needs to be mentioned. They are negative for FDC markers but positive for EBV and SMA [39, 40]. Some authors believed they were related to FDCS [15] and were sometimes categorized as FDCS [41] based on close histogenesis of myofibroblast and follicular dendritic cell, which are both derived from mesenchymal stem cells. It should be noted that fusion negative IMTs lacking ALK and other receptor tyrosine kinases are mostly seen in young adults [42], in contrast to older patients of EBV+ inflammatory GI FDCS.

It was debatable until recently, whether inflammatory FDCS, EBV positive or EBV negative exists in GI tract. Two cases of EBV negative inflammatory GI FDCS [16, 25] and one case of EBV positive inflammatory GI FDCS were reported [27]. Whether EBV negative inflammatory GI FDCS should be classified based on morphology or EBV status is unclear. Based on the recent findings and emerging evidence of inflammatory EBV+ GI FDCS, we favor the latter classification by their EBV status. Recent reports from different groups identified another 11 cases of EBV+ inflammatory GI FDCS, which are all from Southeast Asia with invariable association with EBV [8–10]. Interestingly enough, these tumors arise exclusively from lower GI tract, presenting as polypoid masses in the colon. Although the tumors rarely recur, the mortality rate is not necessarily lower in the group (Table 1). The overall survival is longer in EBV+ inflammatory FDCS patients than that in classic type, but the difference is not statistically significant, probably because of short follow-up time of majority of patients in the former group. Histologically, EBV+ inflammatory FDCS consists of a small number of spindle cells in a background of prominent lymphoplasmacytic infiltrate. Variable degree of nuclear atypia is found, with at least few cells with overt atypia. Cells resembling Reed-Sternberg cells can also be seen [9]. The vessel walls may show hyalinization and fibrinoid change [8, 9].

Currently the pathogenesis of FDCS is not completely understood. It is believed that Castleman disease could be a precursor lesion of classic type FDCS. Follicular dendritic cells in both Castleman disease and FDCS express high levels of epidermal growth factor receptor, which along with its ligands, drives FDC proliferation and survival [43, 44]. It was proposed that FDCS may arise from the dysplastic FDCs in the lymph nodes with Castleman disease [6, 7, 45]. Studies of transcriptional profiles of FDCS reveal additional phenotypical markers [36], suggesting an enhanced transcription program in the transformed neoplasm. In addition to complex cytogenetic karyotypes, the most common genetic alterations seen in FDCS include loss of function mutations in *NF-κB* negative regulatory genes, inhibitory cell cycle control genes [46] and *BRAF* V600E mutation [46, 47]. On the other hand, EBV is associated with pathogenesis of EBV+ inflammatory FDC [48]. CD21, a receptor for EBV, is expressed by FDCs, which enables the entry of oncogenic virus into cell. Presumably as in nasopharyngeal carcinoma [49], EBV infection in follicular dendritic cells (or its stem cells) may cause malignancy transformative activation of Akt and *NF-κB* signalings as well as other oncogenic alteration. Thus it is possible that whether classic or inflammatory EBV+, FDCS has a common pathway of activated *NF-κB* signaling during development. In our unusual case, the patient was found to harbor a *FLCN* germline mutation pathogenic for Birt-Hogg-Dubé syndrome. This is the first report of FDCS in a syndromic patient. It is unclear whether *FLCN* mutation is a driver mutation for FDCS or a bystander. However it is known that FLCN modulates mTOR pathway [50], which functions upstream of Akt and NF-κB signalings.

## Conclusion

FDCS may present with variable histomorphology and immunohistochemical phenotypes. With increasing number of reported cases, it becomes clear that there are two subtypes of FDCS in GI tract, classical type and inflammatory EBV positive type. They display distinctive morphology and clinicopathological features, indicating different pathogenesis in the development. We present the first case of classical subtype of FDCS in a syndromic patient, which may potentially shed lights on the pathogenesis of FDCS. Whether *FLCN* mutation is involved in the growth of FDCS needs to be determined in the future.

## Data Availability

The datasets used and analyzed during the current study are available from the corresponding author on reasonable request.

## Abbreviations

EBV: Epstein-Barr virus
FDC: Follicular dendritic cell
FDCS: Follicular dendritic cell sarcoma
GI: Gastrointestinal
GIST: Gastrointestinal stromal tumor
IMT: Inflammatory myofibroblastic tumor
